# The Influencing Factors of Medical Postgraduates’ Usage Intention Toward Artificial Intelligence–Generated Content Tools in Academic Research: Qualitative Analysis

**DOI:** 10.2196/77928

**Published:** 2026-05-01

**Authors:** Chen Wang, Liu Wang, Xuejiao Zhang, Huiying Qi

**Affiliations:** 1 Department of Health Informatics and Management, School of Health Humanities, Peking University, 38 Xueyuan Rd, Haidian District, Beijing, China, 86 13611019865; 2 Department of Language and Culture in Medicine, School of Health Humanities, Peking University, Beijing, China; 3 School of Nursing, Peking University, Beijing, China

**Keywords:** artificial intelligence tools, medical students, scientific research, grounded theory, influencing factors

## Abstract

**Background:**

The integration of artificial intelligence–generated content (AIGC) tools into academic research offers transformative potential for enhancing productivity and innovation. However, within the highly regulated and ethically sensitive medical context, the use of AIGC is accompanied by significant challenges. Medical postgraduates, as the future vanguard of medical science, play a crucial role in the advancement of digital health, and their intention to use AIGC tools will significantly influence the use of these emerging technologies in medical research. Despite the growing popularity of AIGC tools, there remains a paucity of in-depth understanding of the factors driving or hindering medical postgraduates’ intention to use these tools in academic research. A clear comprehension of these influencing factors is essential to foster the responsible, effective, and sustainable integration of AIGC into medical research.

**Objective:**

This study aimed to systematically explore the key factors influencing medical postgraduates’ intention to use AIGC tools in academic research, with the goal of informing strategies to promote their ethical use and enhance scholarly research capabilities.

**Methods:**

We used a qualitative research design based on grounded theory. Semistructured interviews were conducted with 30 medical postgraduates across diverse specialties, all of whom had prior research experience and familiarity with AIGC tools. Participants were recruited purposively to ensure diverse perspectives. Data analysis followed a systematic coding process to inductively develop a conceptual model, which was further structured and interpreted through the theoretical lens of the Unified Theory of Acceptance and Use of Technology.

**Results:**

Our analysis identified 7 core factors directly shaping usage intention: performance expectancy, effort expectancy, social influence, facilitating conditions, individual characteristics, task characteristics, and technology characteristics. Further analysis revealed that performance expectancy acted as a mediating variable in the relationships between both task characteristics and technology characteristics and usage intention. Additionally, social influence moderated the relationship between task characteristics and performance expectancy. The research findings underscore that, while AIGC tools are valued for assisting daily research tasks, medical postgraduates’ intention to use them in academic research is influenced by technical deficiencies, high cognitive load, and the strict ethical risks and data governance requirements in the medical field.

**Conclusions:**

This study constructs a conceptual model aimed at elucidating the influencing factors of medical graduate students’ intention to use AIGC in academic research. Recommendations derived from the findings include (1) fostering artificial intelligence literacy and critical competency among medical postgraduates; (2) optimizing AIGC tools to better address domain-specific needs, accuracy, and security concerns prevalent in health research; and (3) establishing clear academic supervision and ethical governance mechanisms to ensure responsible use. These measures are essential to harness the potential of AIGC while safeguarding the rigor and integrity of medical academic research.

## Introduction

### Background

As artificial intelligence (AI) technology advances to a new level, artificial intelligence–generated content (AIGC) tools, such as ChatGPT, are emerging. These tools are capable of effectively generating content and comprehending context, offering robust support for users to rapidly and efficiently gain knowledge and enhance the productivity and quality of learning and work. They have also had a significant impact on the development of many industries. Within the realm of academic research, AIGC tools have proven to be instrumental in research design, academic search, data processing, data analysis, and paper refinement, serving as a vital aid for researchers [1]. However, the issues of academic integrity, academic research ethics, and data privacy protection that AIGC tools introduce have also surfaced [2].

Consequently, how to effectively respond to risks and ensure the standardization and sustainability of academic research activities while reasonably using the advantages brought by AIGC has become a crucial area of investigation in the field of AIGC-empowered academic research.

Against this background, scholars have carried out a series of studies on the application of AIGC tools in the field of academic research, including how AIGC empowers various disciplines, how it is applied in different academic research links, and how to deal with the changes brought by AIGC tools. However, during the literature review process, we found that this research has not yet conducted an in-depth analysis of the factors influencing the intention of medical postgraduates to use AIGC tools in academic research. As postgraduates are the future backbone of the research team, cultivating their research abilities is crucial for the development of their respective industries. Exploring the factors influencing their use of AIGC tools in academic research work will help guide them to use AIGC tools reasonably to improve their academic research ability and efficiency. Medical postgraduates are the main force in the realm of medicine, so their attitudes, needs, and behavioral characteristics toward AIGC tools will also affect the sustainable development and promotion of AIGC in the medical field.

### Objective

This study will explore the motives, needs, obstacles, and concerns of medical postgraduates when using AIGC tools in the academic research process; analyze how different factors affect medical postgraduates’ intention toward AIGC tools in academic research; provide a basis for the cultivation of academic research ability of medical postgraduates; and also help promote the optimized application of AIGC tools in the field of medical education and academic research.

### Related Studies

#### Research on the Applications of AIGC Tools in Academic Research

In the realm of academic research, AIGC tools (such as ChatGPT and DeepSeek) are increasingly gaining widespread attention. These tools, with their ability to simulate human intelligence, can efficiently process and generate a wide range of content, offering new possibilities for scientific practice.

Research has shown that ChatGPT can significantly enhance research efficiency by rapidly searching and organizing a vast amount of literature, which can effectively reduce the time researchers spend on collecting materials [3]. Moreover, AIGC tools can provide researchers with new perspectives and pathways for research due to their powerful capabilities for resource acquisition and analysis [4-6]. In academic writing, AIGC tools also play a significant role in improving the standardization and quality of articles [7,8]. In the field of medical research, AIGC tools have demonstrated potential applications in assisting with medical text writing [9], image analysis [10], and disease diagnosis and treatment [11,12].

Despite the significant advantages that AIGC tools provide in academic research, they also contribute to certain limitations and potential risks. Some studies have pointed out that when conducting literature reviews, AIGC tools may lack autonomy and cannot independently generate new ideas [4]. Besides, AIGC tools may fail to effectively distinguish the reliability of information and potentially introduce inaccurate or misleading data, which could pose a threat to research quality and academic integrity [13,14]. Additionally, AIGC tools face multiple challenges, including data bias, information leakage, and ethical issues. Particularly in data analysis, as researchers’ dependence on AI increases, it may further undermine the independence of academic research [15].

In the medical field, academic research involves a large amount of sensitive data, which directly relates to patients’ health and safety. Therefore, ensuring privacy protection and ethical considerations in medical research is particularly critical [2,16]. Given the characteristics of medical research, the precision, credibility, and security of AIGC tools must undergo rigorous testing and evaluation. Consequently, exploring how to reasonably and safely apply AIGC tools in medical research to ensure the accuracy, completeness, and academic integrity of studies, while fully safeguarding patient privacy and data security, has become an urgent and important issue that needs to be addressed.

#### Research on the Factors Influencing the Intention to Use AIGC Tools

The intention to use AIGC is a prerequisite for its widespread application. Scholars have explored the acceptance, adoption, and intention to use AIGC tools through various methods and theoretical models.

Some studies have used the Technology Acceptance Model to reveal the factors influencing different user groups’ intention to use AIGC tools. It has been found that perceived risk, perceived usefulness, perceived ease of use, and attitude toward technology play important roles in the adoption of ChatGPT [17]. Other studies have mentioned that intention to use is significantly influenced by personal ability, social influence, perceived usefulness of AI, enjoyment, trust in AI intelligence, positive attitude, and metacognitive self-regulated learning [18]. Studies based on the Unified Theory of Acceptance and Use of Technology (UTAUT) model have further expanded the research perspective. Relevant studies have shown that performance expectancy, social influence, and effort expectancy significantly affect the intention to use ChatGPT, while facilitating conditions do not have a direct relationship with usage intention [19]. Menon and Shilpa [20] found that the 4 core factors of UTAUT (performance expectancy, effort expectancy, social influence, and facilitating conditions) and 2 extended factors (perceived interactivity and privacy concerns) can effectively explain user interactions with ChatGPT. Tao et al [21], based on the UTAUT model, added 3 factors: perceived risk, resistance bias, and personal innovativeness, and found that these factors are significantly positively correlated with the intention to use AI chatbots.

In addition, research has increasingly focused on the student perspective, analyzing the factors influencing the use of AIGC tools in different educational contexts. Some studies have found that students’ perceived value of ChatGPT significantly affects their intention to use it, with perceived usefulness, perceived enjoyment, and perceived cost being important factors influencing perceived value. Moreover, studies have shown that students with higher levels of AI literacy have a higher acceptance of AIGC tools [22]. A study on medical students’ intention to use ChatGPT in an interdisciplinary programming course analyzed influencing factors from 4 aspects: individual (such as learning motivation), technology (such as tool reliability), information (such as content accuracy), and environment (such as teaching support) [23].

Despite the fact that existing research has analyzed the intention to use AIGC and its influencing factors from multiple angles, previous research has paid less attention to the factors influencing the use of AIGC by medical postgraduates in academic research. As medical postgraduates are the main force in medical research and practice, their acceptance and intention to use AIGC will directly affect the long-term development of AIGC in the medical field. Therefore, this study takes medical postgraduates as the research object and explores their applications of AIGC in academic research and the influencing factors.

## Methods

### Study Design

Currently, research on the factors influencing the use of AIGC tools in academic research activities has yet to establish a mature theoretical model, variables, or scales. Therefore, this study uses the classic grounded theory proposed by Glaser et al [24] as the research methodology. Grounded theory, as an inductive qualitative research approach, is dedicated to constructing robust theories from empirical data. By coding the collected raw data and conducting multiple continuous comparisons, this method enables researchers to maintain a high degree of theoretical sensitivity throughout the evolution of concepts and categories, thereby uncovering the factors that influence the use of AIGC tools by medical postgraduates in academic research.

In accordance with the aforementioned research approach, the study design is based on the following five steps:

1. Based on the pre-established theoretical framework, this study has devised a sampling strategy to ensure the inclusion of medical postgraduates with extensive clinical experience and diverse perspectives.
2. Research participants are capable of providing comprehensive information regarding the phenomenon under investigation, which facilitates researchers’ in-depth understanding.
3. Data collection and analysis will be conducted concurrently until new information no longer contributes to the expansion of the theory, that is, until data saturation is achieved.
4. As theoretical insights become progressively clearer, the data collection strategy may be correspondingly adjusted.
5. Through continuous comparison, integration, and reflection, the study ultimately constructs a theoretical model grounded in empirical data.

### Participant Recruitment

Participants were selected according to the following criteria: (1) postgraduates in medical-related fields with research experience and systematic academic research training; (2) familiarity with and prior use of AIGC tools; and (3) voluntary participation, fluent communication skills, independent thinking ability, and intention to provide detailed information.

Recruitment was conducted through three channels: (1) advertisements posted in WeChat groups and on WeChat Moments; (2) snowball sampling, where initial participants referred other eligible individuals; and (3) outreach via platforms such as Zhihu, Weibo, and Rednote.

The sample size of 30 medical postgraduates was determined based on the principle of “information power,” prioritizing depth and diversity over numerical representativeness. To capture a wide range of experiences, the purposive sampling strategy ensured variation in key dimensions such as gender, disciplinary background (eg, internal medicine, surgery, basic sciences), and stage of research training.

### Data Collection

Semistructured interviews served as the primary data collection method. The interview outline was developed in three stages: (1) drafting based on prior research and the UTAUT theory; (2) revision through discussions with 2 researchers experienced in AIGC tool application; and (3) pilot testing with 5 users, followed by refinement based on feedback.

The UTAUT was used as the foundational framework for this study. Although UTAUT was originally developed to explain technology acceptance in organizational settings [25], its core constructs, including performance expectancy, effort expectancy, social influence, and facilitating conditions, are broadly applicable to individual-level technology adoption across various domains. In the context of academic research, these constructs align closely with the key drivers of technology use among postgraduate researchers, who must balance efficiency, effort, peer influence, and institutional support when integrating new tools into their workflows.

Each interview lasted 30 to 40 minutes. With participants’ consent, all interviews were audio-recorded. Recordings were then transcribed verbatim to form the raw textual data for analysis. All participants provided informed consent and agreed to the anonymous use of their quotes.

### Data Analysis

Prior to the coding of the interview data, a rigorous screening process was conducted based on two main criteria: (1) the clarity of the descriptions and (2) the relevance of the descriptions to the topic. This process was completed by CW and HQ, and they also integrated similar descriptions for subsequent analysis. To ensure comprehensive familiarity and sensitivity to the data, the researchers repeatedly read through the transcripts and descriptions, thereby minimizing the risk of losing key information. This study followed the 3-step coding process (open coding, axial coding, and selective coding) proposed by Venkatesh et al [25] for qualitative analysis and assessed the stability of the coding results through a theoretical saturation test. The specific analysis process is as follows:

1. Open coding: The raw data were initially organized and semantically deconstructed. Basic conceptual units were extracted through line-by-line coding. Researchers employed the constant comparative method to conceptually annotate the original sentences, forming an initial category system through multiple rounds of classification and integration.
2. Axial coding: Based on the primary categories formed from open coding, category relational analysis was used to identify potential logical relationships. Through dimensional comparison and attribute induction, a hierarchical structure among categories was established, ultimately constructing a main category framework with explanatory logic.
3. Selective coding: Theoretical integration was conducted around the core category, systematically sorting out the logical relationships between the main categories and the core category. A theoretically consistent framework was formed through storyline analysis.
4. Theoretical saturation verification: Saturation was confirmed through an iterative process of data collection and analysis. After initial coding of the majority of interviews, a sample addition method was employed, where approximately 10% of new transcripts were sequentially analyzed. Theoretical saturation was deemed reached when these new samples no longer yielded novel categories, properties, or significant relationships but merely reinforced the existing coding structure. The entire analytical process was strengthened by researcher triangulation (independent coding and consensus discussions) and peer review to enhance the reliability and validity of the findings.

LW and XZ coded the interview texts separately, and any coding discrepancies throughout the process were resolved through discussion between LW and XZ. If consensus could not be reached, CW acted as an arbitrator to determine the coding results. Data analysis was supported by NVivo 11.0 software.

### Ethical Considerations

The participants of this study were master’s and doctoral students in medical schools. The interviews were conducted face-to-face, and informed consent was obtained. Before the interviews, the contents of the informed consent form and the outline of the interview were read to the participants, and all participants provided signed the informed consent form. Participants were compensated with 50 RMB (US $7.33) for their time. The Institutional Review Board of Peking University approved the informed consent form and the full research proposal (IRB00001052-24025).

## Results

### Study Sample

The participants in this study consisted of 30 medical postgraduates, including 18 (60%) female participants and 12 (40%) male participants from diverse medical majors. All participants had academic research experience and were familiar with AIGC tools, with prior usage experience. Table 1 shows the demographic characteristics of all participants.

**Table 1.** Demographic characteristics.

Category	Value, n (%)
Gender	
Male	12 (40)
Female	18 (60)
Specialty	
Clinical medicine	10 (33.3)
Basic medicine	6 (20)
Preventive medicine	6 (20)
Pharmaceutical sciences	5 (16.7)
Nursing science	3 (10)
Frequency of AIGC^a^ tool usage	
Daily use	20 (66.7)
Weekly use	8 (26.7)
Use when need	2 (6.7)
Academic research experience	
1‐2 years	2 (6.67)
2‐3 years	7 (23.3)
3 years and more	21 (70)

a AIGC: artificial intelligence–generated content.

### Influencing Factors Model Framework of Usage Intention

**Figure 1.**
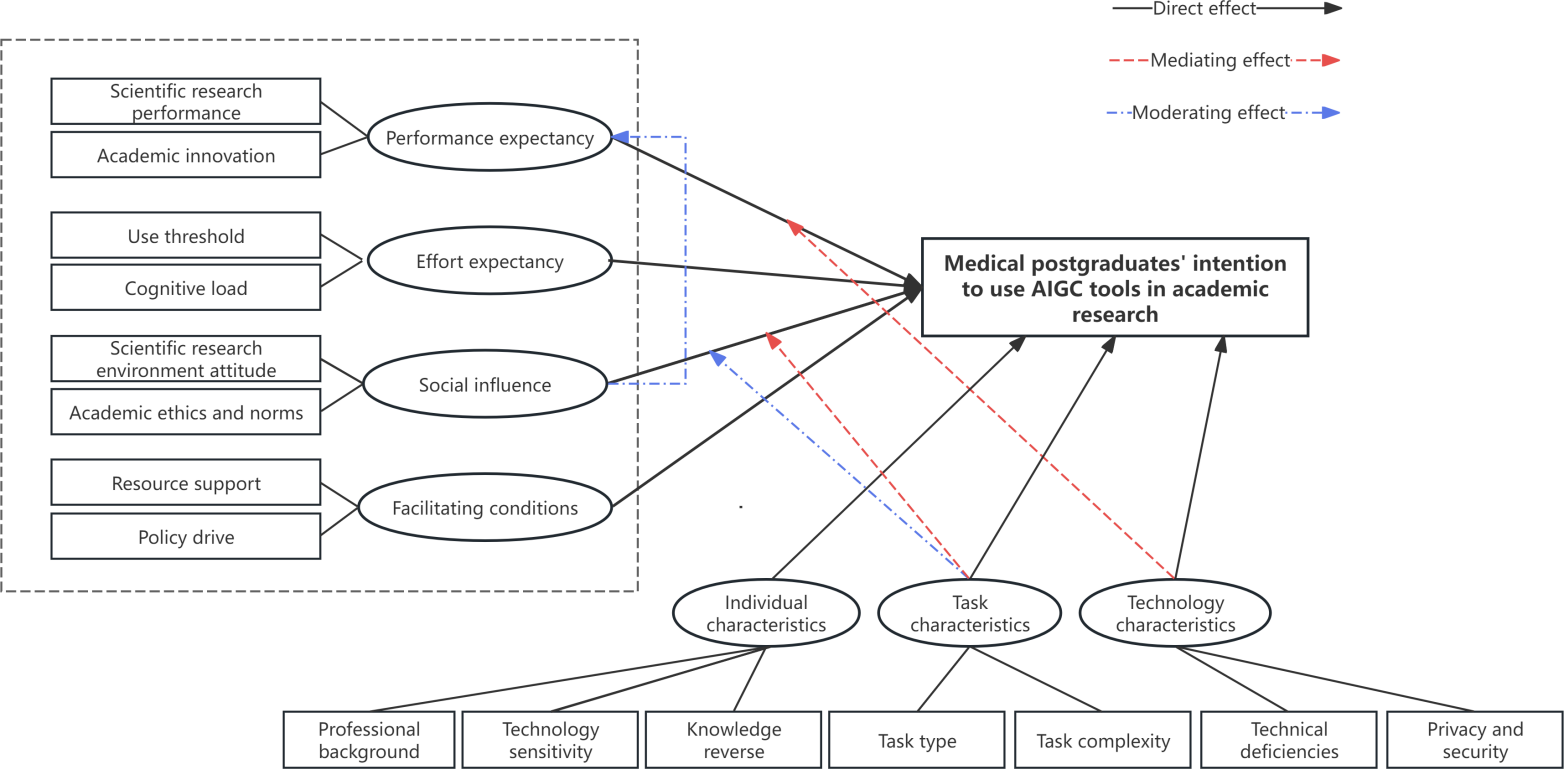
Model framework for the influencing factors of medical postgraduates’ usage intention of artificial intelligence–generated content (AIGC) tools in academic research.

Based on grounded theory and integrated with the UTAUT model theoretical framework to enhance theoretical explanatory logic, this study constructed a usage intention influencing factors model for medical postgraduates’ use of AIGC tools in academic research. The model comprehensively considers 7 dimensions: performance expectancy, effort expectancy, social influence, facilitating conditions, individual characteristics, task characteristics, and technology characteristics (Figure 1), aiming to holistically reveal the key factors affecting medical postgraduates’ effective use of AIGC tools for academic research activities.

### Performance Expectancy

Performance expectancy refers to an individual’s anticipated work effectiveness and benefits after using a particular tool or technology [26], which consists of 2 key components: academic research performance and academic innovation.

In terms of academic research performance, AIGC tools can assist users in literature collection, materials reading, data processing, and paper writing, reducing time costs and enhancing research quality. Moreover, AIGC tools’ capabilities in text polishing and academic translation can enhance article standardization and scientific rigor. These functions enhance users’ performance expectancy, thereby increasing medical postgraduates’ intention and frequency of using AIGC tools.

*When writing articles, my expressions might not be professional enough and the results may not be ideal due to a lack of extensive reading of a large number of literature. In this regard, using AIGC can help me improve my writing quality.*

[P 2]

Regarding academic innovation, AIGC tools can help researchers expand research ideas, discover new research questions, and meet personalized research needs. The study found that over half of respondents recognized AIGC tools’ role in expanding ideas in interdisciplinary or emerging research fields. However, some users pointed out limitations in providing innovative solutions, and the feasibility of generated solutions requires improvement. This indicates that AIGC tools’ innovation capability needs optimization to better meet the innovation demands of high-level academic research.

*If I want to write a certain type of paper, I usually ask it to help broaden my ideas and perspectives. It might provide me with some new sparks.*

[P 15]

### Effort Expectancy

Effort expectancy refers to the perceived difficulty level that users associate with using a particular system [25], consisting of 2 concepts: use threshold and cognitive load.

Use threshold includes AIGC tools’ interface design clarity and operational simplicity and intuitiveness, which influence user experience. Well-designed and easy-to-use AIGC tools can significantly enhance usage intention. Conversely, requiring users to repeatedly familiarize themselves with tool functions increases operational burden and reduces efficiency, thereby diminishing the desire to continue use.

*The main obstacle to using AIGC is the setting of prompts for questions. During the questioning process, AIGC often misinterprets your meaning, and you have to keep making adjustments to get what you want.*

[P 11]

Cognitive load refers to the attention and cognitive efforts medical postgraduates invest when using AIGC tools, encompassing energy expended in understanding operational logic, screening and judging generated content, and effectively integrating generated content with research projects. Cognitive load increases with operational complexity or content processing difficulty. When the cognitive load exceeds the tolerable range, it reduces medical postgraduates’ intention to use AIGC tools.

*After using AIGC to generate code, I have to spend a lot of time debugging and optimizing it before applying results to my research project. This process is more complex than expected, which wears down my patience and enthusiasm, so I rarely use it now.*

[P 23]

### Social Influence

Social influence refers to the degree to which users are influenced by the surrounding environment and people when using technology or services [25]. This category comprises 2 concepts: academic research environment attitude and academic ethics and norms.

In the academic research environment, team members’ acceptance of new technologies and their recognition of usage outcomes directly affect individuals’ usage intention. Conversely, other respondents noted that due to supervisors’ or team members’ reservations about using AIGC tools, they reduced their usage frequency.

*Many labs around me purchase AIGC tool memberships for their students. After trying them out, we recommended AIGC tools to each other and generally think that they were very practical.*

[P 3]

Moreover, academic ethics and norms constitute an important dimension. When using AIGC tools, respondents may worry about potential risks related to academic integrity and copyright. Additionally, regulations from academic research ethics committees or academic journals regarding AIGC use can constrain usage intention. Although some respondents believe that using AIGC tools for noncore content creation, such as literature reviews, is less likely to cause academic disputes, most still avoid or cautiously use directly generated content in paper writing due to concerns about intellectual property rights and plagiarism issues.

*I felt that it is still in a gray area in terms of research ethics. We are worried about the ethics of using AIGC tools and what ethical usage guidelines exist. Special attention must also be paid to academic integrity.*

[P 6]

### Facilitating Conditions

In this study, facilitating conditions refer to the social resource support and policy promotion for AIGC tools.

This study found that insufficient resource support restricts the widespread use of AIGC tools among medical postgraduates. Interview results reveal that lack of resource support manifests in 2 aspects: first, the absence of specialized courses makes it difficult for users to obtain systematic guidance and training, affecting their ability to master and use AIGC tools. Second, the low level of resource popularization results in limited coverage of AIGC tools.

*I learned about a database at our school last year. They were trying to use embedded AIGC to assist with writing. But it hasn't been implemented yet because the school needed to spend extra money to purchase it.*

[P 10]

In terms of policy support, the main problems include the imperfection of laws and regulations and restrictions from ethical norms. Relevant laws on AI use are still being improved, meaning users face certain legal risks when using AIGC tools. In the medical field, policy attitudes toward AIGC tools are more cautious. In addition, using AIGC to assist in creation may lead to intellectual property infringement risks, further limiting the application of AIGC tools.

*The Medical AI Clinical Application Guidelines issued by the National Health Commission last year particularly emphasized data desensitization. When using AIGC for case analysis, we must pass hospital ethics review to avoid crossing the red line.*

[P 26]

### Individual Characteristics

Individual characteristics refer to medical postgraduates’ personal traits influencing their use of AIGC tools. This category includes 3 concepts: professional background, technology sensitivity, and knowledge reserves.

Interview results show that academic background affects respondents’ enthusiasm and necessity for using AIGC tools. For instance, clinical postgraduates, who need to keep up with cutting-edge technologies, are more inclined to use AIGC tools for literature search and new knowledge acquisition. In contrast, postgraduates majoring in traditional Chinese medicine focus more on traditional textbooks and ancient medical texts, thus having a relatively lower usage frequency of AIGC tools. This difference indicates that academic background not only determines the need for AIGC tools but also affects acceptance and frequency.

*As a pharmaceutical science student, I believe that AIGC tools are very useful in predicting drug interactions, generating individualized medication regimens, and analyzing multidimensional pharmacovigilance data.*

[P 30]

Moreover, respondents with higher sensitivity to technology are more willing to try using AIGC tools and can better use their functions.

*I have a keen interest in AI technology, so I always try out new AI tools.*

[P 1]

However, over half of respondents mentioned that when answering medical professional questions, AIGC tools may lack accuracy and comprehensiveness, which requires further verification. Consequently, individuals’ knowledge reserves significantly impact AIGC tool use. Students with rich knowledge reserves can more efficiently use the information provided by AIGC tools and accurately judge and verify results. Conversely, students with insufficient knowledge may find it difficult to distinguish generated information and lose interest in using AIGC tools.

*Generally, when I ask exploratory questions, I already possess some understanding or background knowledge in that field. When GPT provides an answer, I can judge its reliability based on my existing knowledge.*

[P 9]

### Task Characteristics

Task characteristics refer to research task traits that influence medical postgraduates’ use of AIGC tools, directly affecting usage intention and indirectly influencing it through performance expectancy and social influence. This category is divided into 2 concepts: task type and task complexity.

Medical postgraduates selectively use AIGC tools to enhance work efficiency based on academic research task type and difficulty. Most respondents tend to use AIGC tools for language expression optimization tasks, as they can effectively improve text quality and assist in presenting results. However, when confronted with highly specialized academic research tasks like R language programming, some respondents indicated that they would not prioritize AIGC tools. This is due to their subpar performance in such scenarios and sometimes failing to complete tasks, thus struggling to meet academic research needs.

*My main use of AIGC functions related to academic research is writing, not generating text, but letting it help polish my writing. Additionally, I use it to assist with literature search, such as quickly grasping the key points, experimental methods, and conclusions of a paper, saving significant time.*

[P 18]

Moreover, respondents are more inclined to use AIGC tools for simple tasks (such as literature search and translation), which can significantly improve efficiency and reduce repetitive work. However, for tasks involving creativity or high specialization, the output of AIGC tools may not be satisfactory, which makes medical postgraduates more cautious in their use.

*In scientific illustration, AI remains deficient, particularly in medical illustration which demands meticulous attention to detail. AI appears currently incapable of fully replacing human operation in this field.*

[P 19]

It is evident that simple or specific tasks directly prompt medical postgraduates to use AIGC tools. Moreover, task type and complexity affect performance expectancy regarding task success. Simple tasks, due to minimal difficulty, lead medical postgraduates to anticipate that AIGC tools can complete them with ease and efficiency. For specific research tasks, medical postgraduates evaluate task characteristics to determine whether they fall within the scope where AIGC tools can effectively function. This evaluation forms performance expectancy regarding task objective achievement, subsequently enabling them to assess the applicability of AIGC tools and decide whether to use them.

Based on varying performance expectancy arising from different task characteristics, medical postgraduates prioritize research tasks where the use of AIGC tools can yield expected benefits, such as efficient completion and high-quality outcomes. Thus, task characteristics influence performance expectancy, which in turn affects usage intention. Performance expectancy plays a mediating role between task characteristics and usage intention.

*I had extensive sarcopenia data but didn't know how to analyze it. I threw the problem and data at the AIGC tool and it listed many research directions. I felt these directions could provide inspiration, ultimately helping me determine my research direction.*

[P 7]

In addition, depending on task characteristics, social influence modulates its effect on performance expectancy, further influencing the intensity of medical postgraduates’ use of AIGC tools in academic research. For tasks with varying characteristics, positive social influences (eg, strong recommendations from supervisors, successful peer experiences, and extensive media coverage) can reinforce medical postgraduates’ perception that AIGC tools enable efficient and high-quality task completion or alleviate concerns about using such tools. This strengthens the positive impact of task characteristics on performance expectancy, thereby promoting the use of AIGC tools for research among medical postgraduates. Conversely, negative social influences (eg, authoritative voices highlighting ethical risks or shared experiences of errors and malfunctions) can undermine medical postgraduates’ confidence in AIGC tool reliability. These influences raise doubts about the quality and efficiency of using AIGC for research tasks, subsequently lowering performance expectancy, which may lead to reduced frequency and scope of using AIGC tools in academic research.

*When discussing ideas generated by ChatGPT with clinical classmates, they mentioned that these ideas might not be applicable in clinical settings and could not accurately satisfy clinical needs. Therefore, I feel that the ideas provided by ChatGPT are more academic, but I would not widely use them in clinical practice.*

[P 29]

### Technology Characteristics

Technology characteristics refer to AIGC tool traits that influence medical postgraduates’ use of AIGC tools, consisting of 3 key components: technical deficiencies, privacy, and security.

Regarding privacy protection, some respondents explicitly expressed concerns about privacy risks associated with AIGC tools and demonstrated caution in actual use.

*If I send some patient data to it, I’m unsure how it will use this data, or whether others might access it for unknown purposes. I’m uncomfortable with that.*

[P 20]

In terms of technical deficiencies, interview results show that AIGC tools perform poorly in complex mathematical calculations, updating knowledge in specialized fields, and maintaining coherence in multiturn dialogues.

*For highly specialized knowledge, GPT may not provide satisfactory responses and lacks a particular preference for medical terms. In fact, I believe it still falls short in professional domains such as cohort study, control design, and epidemiology.*

[P 12]

It is evident that technology characteristics serve as external factors influencing medical postgraduates’ use of AIGC tools for research, directly shaping usage intention. When privacy concerns arise or AIGC tools fail to ensure generated output quality due to inherent technical flaws, medical postgraduates tend to avoid using such tools. Additionally, regarding indirect influence, performance expectancy plays a mediating role between technology characteristics and usage intention. Variations in the accuracy, security, and other technological attributes of AIGC tools affect respondents’ anticipated outcomes regarding their ability to achieve research objectives with these tools. If technology characteristics lead medical postgraduates to believe that tools can effectively facilitate research and deliver expected results, performance expectancy rises, prompting them to choose AIGC tools. Conversely, if technology characteristics undermine their confidence in achieving research goals, performance expectancy declines, thereby suppressing usage intention.

*When looking up background information and general theories, GPT provides good responses. However, for the latest research findings in highly specialized academic papers, the information provided is sometimes inaccurate and insufficient. Then I'll think relying on it to finish research tasks might not yield good results. So, I prefer looking up literature myself, as it is more reliable.*

[P 16]

## Discussion

### Key Findings

Through semistructured interviews and grounded theory analysis, this study systematically explored the motivations, needs, obstacles, and concerns of medical postgraduates in using AIGC tools for academic research. The findings indicate that their motivation primarily stems from performance expectancy, such as improving research efficiency, assisting in paper writing, and expanding research ideas. Obstacles to use include effort expectancy (eg, high use thresholds and cognitive load) and deficiencies in technology characteristics (eg, insufficient coverage of professional knowledge and poor coherence in multiturn dialogues). Additionally, academic ethics and norms, including academic integrity, data privacy, and copyright risks, constitute significant social concerns that considerably constrain their intention to use these tools.

The study further identified 7 key influencing factors that directly shape medical postgraduates’ AIGC usage intention: performance expectancy and effort expectancy are the core internal drivers, while social influence and facilitating conditions serve as crucial external environmental and supporting factors that promote the functioning of the aforementioned elements by providing external support and convenience; individual characteristics, task characteristics, and technology characteristics differentially influence tool selection, depth of use, and trust.

Regarding the underlying mechanisms, the study found that performance expectancy mediates the relationship between task and technology characteristics and usage intention. This indicates that the features of tasks and technology indirectly influence usage intention by shaping performance expectancy. Furthermore, social influence moderates the relationship between task characteristics and performance expectancy, ultimately affecting the intensity with which medical postgraduates use AIGC tools in academic research. Based on these findings, the study integrates individual, task, and technology factors into the UTAUT theoretical framework, constructing a more comprehensive and discipline-specific model to explain AIGC usage intention among medical postgraduates.

### Influencing Factors

Consistent with prior literature [20], performance expectancy is a primary determinant of usage intention. AIGC capabilities in literature processing, data organization, and manuscript refinement enhance research efficiency and output quality, thereby reinforcing usage intention. However, these tools’ capacity to support high-level academic innovation remains limited, highlighting a need for further technological optimization [27,28].

Effort expectancy is a critical determinant of usage intention [29]. Well-designed, user-friendly tools significantly enhance usage intention, while repeatedly familiarizing oneself with functions or adjusting prompts increases operational burden and reduces efficiency, thereby diminishing continued usage intention. Due to professional terminology and structured logic characteristics in medical research, medical postgraduates typically face higher initial learning thresholds [30,31]. In contrast, students in fields such as computer science or humanities may find AIGC tools more aligned with their natural language processing or creative writing tasks [32,33].

Social influence significantly affects usage intention [34,35]. Within research teams, usage is shaped by the attitudes of peers and supervisors; endorsement fosters usage, while skepticism acts as a deterrent. Beyond interpersonal influence, strict adherence to academic integrity and copyright standards constrains the use of AIGC in formal writing. Furthermore, the medical research environment is characterized by rigorous ethical oversight and institutional review boards, exerting stronger normative pressure compared to the more flexible, innovation-driven cultures of engineering and design [36-40].

Facilitating conditions influence usage intention [41,42]. Key constraints include insufficient resource support and imperfect policies. Risks related to data privacy and intellectual property further restrict the usage in formal research. Moreover, medical postgraduates face more stringent data governance and ethical oversight (eg, Health Insurance Portability and Accountability Act, General Data Protection Regulation) [43,44], creating higher compliance barriers compared to fields like engineering where resource access and policy environments are more supportive [16].

Individual characteristics significantly influence AIGC usage intention. Consistent with prior research, academic background shapes perceived necessity and frequency of use, while higher technology sensitivity correlates with increased usage intention and functional mastery [45,46]. A user’s knowledge reserve is critical for effectively verifying and utilizing AIGC outputs; insufficient knowledge can undermine trust and discourage continued use [47]. Notably, the professional identity of medical postgraduates instills a distinct skepticism compared to creative disciplines, prioritizing diagnostic accuracy and evidence reliability over generative convenience [48].

Task characteristics directly impact usage intention [49,50] and indirectly affect it through performance expectancy and social influence. Users are more inclined to use AIGC in routine tasks like literature search and language polishing, while remaining cautious in creative or highly specialized tasks. Additionally, unlike nonmedical disciplines, medical research tasks often involve multimodal data integration, longitudinal study designs, and strict ethical protocols, but AIGC tools’ limited capabilities in areas such as clinical trial design [51] or diagnostic image interpretation [52] inhibit usage intention.

Technology characteristics directly impact usage intention [46,53] and indirectly affect it via performance expectancy. Limitations such as insufficient domain knowledge depth, complex calculation errors, and data privacy risks directly limit tool use and reduce performance expectancy, prompting users to adopt cautious strategies like data anonymization or selective avoidance. Notably, the strict patient confidentiality and regulatory requirements in health care environments make privacy issues more prominent, making medical postgraduates more inclined to use localized or secure AIGC solutions [54,55].

### Measures and Suggestions

#### Fostering AI Literacy Among Medical Postgraduates

To effectively navigate the opportunities and risks presented by AI technologies in the medical field, prioritizing the enhancement of AI literacy among medical postgraduates is imperative [56]. Consequently, strengthening AI literacy education and ensuring the regulated and effective use of AIGC tools are critical objectives. On one hand, practical competencies in using AIGC tools should be cultivated through the development of AI-related curricula and the integration of open educational resources. Specific focus areas include mastering prompt engineering and critically evaluating information credibility; furthermore, engagement in AIGC-assisted medical research projects can deepen students’ understanding of AI applications [31,57]. On the other hand, educators must guide postgraduates to reflect on the societal implications of AIGC [58,59]. It is essential to foster a profound understanding of ethical principles, including transparency, fairness, nonmaleficence, accountability, and privacy protection, and to clarify liability attribution in AI-involved medical decision-making to ensure compliance with medical ethical standards.

#### Optimizing AIGC Service

Our findings indicate that AIGC usage intention among medical postgraduates is impeded by information inaccuracies, data latency, operational complexity, and security concerns. To enhance research utility, AIGC tools could integrate medical knowledge graphs and develop specialized large models to improve domain-specific comprehension. AIGC connection with literature databases enables rapid screening and summary of medical literature, assisting medical postgraduates in staying up to date with cutting-edge research. Moreover, integrating AIGC tool functions and simplifying processes reduces usage barriers and cognitive load. Technical measures like encryption and anonymization protect data security, ensuring ethical compliance and data security [60].

#### Establishing an Academic Research Supervision Mechanism

While promoting AIGC technological innovation, regulatory principles balancing encouragement of innovation and prudent evaluation should be adopted, implementing tiered regulation based on application scenarios and risk levels in medical research. For AIGC applications involving core data, implementing identity verification and generation record trails is essential to ensure traceability and patient privacy. To prevent academic misconduct, institutions should enforce strict ethical reviews and mandate the disclosure of AIGC use in research outcomes [61]. Furthermore, using AI detection tools to screen research results and identify violations, while developing and refining industry standards and regulations to clarify application boundaries, responsible entities, and regulatory requirements, provides action guidelines for medical researchers, thereby enhancing the role of AIGC tools in medical research.

### Limitations

This study has certain limitations. On the one hand, the sample size is relatively small and is limited to medical postgraduates. On the other hand, while the proposed influencing factor model is theoretically plausible, it remains untested through systematic empirical validation and statistical verification, which constitutes a key limitation of this study.

### Conclusion and Future Research

This study adopts the grounded theory qualitative research paradigm for analysis and constructs an influencing factor model of medical postgraduates’ usage intention regarding AIGC tools in academic research based on the UTAUT model. The logical relationships and mechanisms of the influencing factors are analyzed, thereby deepening the understanding of medical postgraduates’ experiences and perceptions of AIGC tools and addressing gaps in the existing literature. The research results indicate that performance expectancy, effort expectancy, social influence, facilitating conditions, individual characteristics, task characteristics, and technology characteristics directly or indirectly affect the usage intention of medical postgraduates toward AIGC tools in academic research.

To support the responsible and effective integration of such emerging technologies into medical research ecosystems, we propose targeted strategies: fostering AI literacy among medical postgraduates, optimizing AIGC services to meet domain-specific needs, and establishing robust academic supervision mechanisms. These recommendations aim to provide practical guidance for enhancing the constructive role of AIGC in advancing medical research while upholding academic rigor and ethical standards.

Considering the limitations of existing research, future research could include more participants to enhance the general applicability of the findings. On the other hand, the study could incorporate questionnaire data and experiments to combine quantitative and qualitative methods, gather richer data, and analyze more deeply, validating mediating and moderating effects and clarifying the specific mechanisms behind them.

## Abbreviations

AI: artificial intelligence
AIGC: artificial intelligence–generated content
UTAUT: Unified Theory of Acceptance and Use of Technology
